# SpeciMate: Improving metadata extraction from digitised biological specimens

**DOI:** 10.3897/BDJ.13.e160553

**Published:** 2025-07-31

**Authors:** Alan Stenhouse, Peter H. Thrall

**Affiliations:** 1 CSIRO, Canberra, Australia CSIRO Canberra Australia

**Keywords:** specimen, digitisation, metadata, AI, software application, data curation

## Abstract

**Background:**

The digitisation of natural history collections represents a critical step towards preserving and increasing accessibility to valuable scientific data. Despite their fundamental importance to taxonomy, ecology and conservation, the world’s natural history collections remain underutilised due to the labour-intensive process of extracting metadata from specimen labels.

**New information:**

This paper describes SpeciMate, a software application that uses a human-AI collaborative approach to accelerate the extraction of metadata from digitised specimen images. The system leverages artificial intelligence web services including optical character recognition (OCR), automated translation and large language and multimodal models (LLMs) to extract structured metadata, while requiring human expertise for prompt engineering and data curation. We describe the application's architecture, functionality and workflows, which enable effective processing of various specimen types including herbarium sheets and insect slides. Our trials indicate that this tool significantly improves the efficiency of metadata extraction while maintaining high data quality. The combination of automated AI processing with human supervision and refinement represents a promising approach to accelerating the digitisation and databasing of natural history collections, thereby enabling broader access to these invaluable resources for research, education and conservation efforts.

## Introduction

Natural history collections worldwide house an estimated 2-4 billion specimens, representing an irreplaceable scientific resource that documents Earth's biodiversity across time and space (Ariño 2010). Despite their fundamental importance, less than 20% of these specimens are readily discoverable or accessible online (Nelson and Ellis 2019), highlighting a critical gap between their potential and realised utility. While these collections have traditionally served as the cornerstone for taxonomic and systematic research, their significance extends far beyond such applications. Specimen collections and their associated data have proven instrumental in addressing global challenges ranging from conservation and biosecurity to human health and climate change (Funk 2018, Meineke et al. 2019).

The digitisation of natural history collections has evolved significantly over the past two decades, with various approaches developed to address the challenges of metadata extraction from specimen labels. Early efforts focused primarily on manual transcription, which, while accurate, proved prohibitively time-consuming and resource-intensive for large-scale implementation (Nelson and Ellis 2019). Crowd-sourcing platforms emerged as a promising solution to scale up digitisation efforts. Initiatives such as DigiVol (https://volunteer.ala.org.au) (Flemons and Berents 2012) and Notes from Nature (Hill et al. 2012), which runs on the Zooniverse (https://www.zooniverse.org/) platform, successfully mobilise volunteers to transcribe specimen data. While these platforms have processed millions of specimens, they still require significant project management overhead, volunteer training and quality control measures to ensure data accuracy (Nelson et al. 2015, Alzuru et al. 2016).

A range of semi-automated methods using optical character recognition (OCR) have also been developed to accelerate the digitisation process. Systems such as SALIX (Barber et al. 2013), Inselect (Hudson et al. 2015), ALICE and others (Price et al. 2018, Dupont et al. 2020) explored novel imaging systems before applying OCR to specimen labels followed by parsing algorithms to extract structured data. More recent approaches have combined improvements in computer vision and natural language processing models using pipelines to improve metadata extraction (Owen et al. 2020, Hardisty et al. 2022, Guralnick et al. 2024). Transformer-based models like BERT and more recent large language models (LLMs) using generative pre-trained transformer (GPT) architectures have shown great potential in named entity recognition tasks for biodiversity data (Alzuru et al. 2020, Abdelmageed et al. 2022, Turnbull et al. 2024). These often use model fine-tuning to achieve good results, which requires technical expertise and carefully selected training data (Dagdelen et al. 2024). LLMs have recently been explored for flexible entity extraction in a variety of industries (Yu et al. 2022, Wang et al. 2023, Keloth et al. 2024, Whittaker and Kitagishi 2024) and within the natural history collections specimen digitisation domain (Weaver et al. 2023, Takano et al. 2024). These solutions demonstrate the interest and significant potential in this approach.

Our work has resulted in an easy-to-use application which incorporates diverse AI models, including LLMs, such as OpenAI’s GPT-4 and others, for no-shot named entity recognition of OCR text combined with an interactive human-AI collaborative workflow specifically designed for natural history collections. Unlike systems requiring extensive training data, custom model development and technical expertise to install and update software libraries, our application leverages readily available AI services to achieve high-quality metadata extraction across diverse specimen types and label formats, making it accessible to collections with limited technical resources and expertise. Through iterative natural language prompt development or “prompt engineering” (Arawjo et al. 2024, Lee et al. 2025), specimen metadata can be extracted and formatted into consistent output for all target entities, including often problematic, but important items such as locality and habitat. While errors can still occur, during both OCR and entity extraction processes, especially when dealing with problematic handwriting and overlapping labels, these highlight the continued importance of human curatorial expertise.

This paper introduces SpeciMate, an open-source executable application that employs a human-AI collaborative approach to metadata extraction from digitised specimen images. SpeciMate integrates publicly available AI services for OCR, translation and entity extraction with interactive interfaces for human input and curation. In the following sections, we provide an overview of the architecture and functionality of SpeciMate, discuss our observations of its use with herbarium and insect specimens and outline potential future directions for development.

## Project description

### Design description

**Application Description**

**Overview**

SpeciMate is an open-source stand-alone application developed using LiveCode (version 9.6.8 and above), a cross-platform development environment that enables deployment on both Windows and macOS systems without the need to install other software libraries. The application is designed to be user-friendly and accessible to collection staff without requiring specialised technical knowledge, while providing powerful capabilities for metadata extraction and curation.

Readily available generalised AI models are used for processing, ensuring these services are available to all, without needing to train or finetune models, which would require resources and knowledge that many collections do not have. The application integrates with web-based AI services through their respective Application Programming Interfaces (APIs), including Google Vision for OCR, Google Translate for translation and OpenAI's large language and vision models for metadata entity extraction. While these services require usage-based payments, this approach leverages powerful AI capabilities without requiring users to install or maintain complex AI infrastructure locally. The application automates and coordinates the OCR, translation and NER processes and can process multiple images concurrently to increase performance, significantly reducing the time and effort required to digitise large collections. Intermediate processed data from the OCR and LLM processes are stored in JSON formatted files in the same folder as the specimen images.

The SpeciMate workflow consists of three main phases:

- *Project Setup and Prompt Engineering*: Users select a folder of specimen images, define parameters and iteratively refine prompts for the AI models to optimise metadata extraction using a sample of specimen images.
- *Batch Processing*: The application processes multiple images automatically, applying OCR, translation (if needed) and metadata extraction according to the defined parameters.
- *Interactive Curation*: Users review and refine the extracted metadata through an interactive interface, with assistance from integrated tools for verification.

**Main Processing Capabilities**

SpeciMate uses the Google Vision API to extract text from specimen images. The process accounts for various text formats, orientations and qualities, including typed and handwritten text and provides the foundation for the next steps. For specimens with labels in foreign languages, SpeciMate offers automatic translation through Google Translate services. As the OCR process also detects when foreign language text is present, if a language is present in significant proportions (a threshold of > 15% of the total text), then it is sent for translation. Users can select to use the translated text for subsequent metadata extraction or not, according to their requirements.

SpeciMate extracts structured metadata from unstructured label text using large language models (LLMs). The application currently accesses OpenAI models, ranging from GPT-4.1 to GPT-4o-mini, with the option to use multimodal models that can perform multiple processes on the images simultaneously. Models from other sources are supported provided they follow the openAI API call and response formats.

A key to effective entity extraction are the prompts that are supplied to an LLM, along with the data. The prompt provides instructions and context for the LLM to assist it in carrying out the task effectively. Other parameters often set when using a LLM API, such as *temperature* (which determines the randomness of token or word selection), are also important for effective entity extraction, but these are mostly hidden from the user, as changing them would likely degrade performance. For example, a lower sampling temperature makes responses more predictable, while a higher temperature introduces more randomness, which is undesirable here. Prompts in SpeciMate consist of several components which are combined with the OCR and/or translated text data during processing. These components include:

- *Role*: Contextual information that sets the stage for the LLM;
- *Prompt*: Detailed instructions on the extraction task;
- *Columns*: Definition of the metadata fields to extract.

To help with the variety of specimen types and data that might be extracted, as well as to assist experimentation and version control, SpeciMate has a prompt management system, which allows users to define, customise and save prompts for different specimen types. The prompt engineering process is iterative, allowing users to test prompts on subsets of images, check the results and refine the prompt details as necessary, before proceeding with batch processing. This human-AI collaboration during project setup is crucial for optimising the performance of the metadata extraction process for each set of specimens.

As part of the prompt information, users can also specify formatting preferences for extracted data (e.g. date formats, capitalisation), provide examples for the AI model and include inferencing instructions for handling missing information. See Table 1 for an example of the prompt details for a set of herbarium specimens.

SpeciMate offers the option to use multimodal AI models that can process images directly, potentially performing OCR, translation and entity extraction in a single pass. This approach may be useful for specimens with unusual formats where the multimodal model can use the spatial context of labels to improve results. Multimodal models may also be useful for handling non-textual data such as pictograms for gender symbols or other specialised notations. Note, however, that these multimodal models currently do not usually perform as well as specialised OCR models and do not provide translation for as many languages as more specialised models.

Following batch processing of specimen image sets, each specimen can be curated using a dedicated specimen metadata curation screen, which allows the user to check and edit the results while viewing the specimen image.

In addition to single specimen images, SpeciMate can also process multiple specimens from a single image, such as a tray of insect slides. It can also process form-based tabular data. In these cases, the resulting dataset is presented in a tabular metadata screen where curation and editing can take place while viewing the original image. Single specimen images have a dedicated curation form for editing the metadata and checking using various tools.

**User Interface and Workflow**

**Figure 1.**
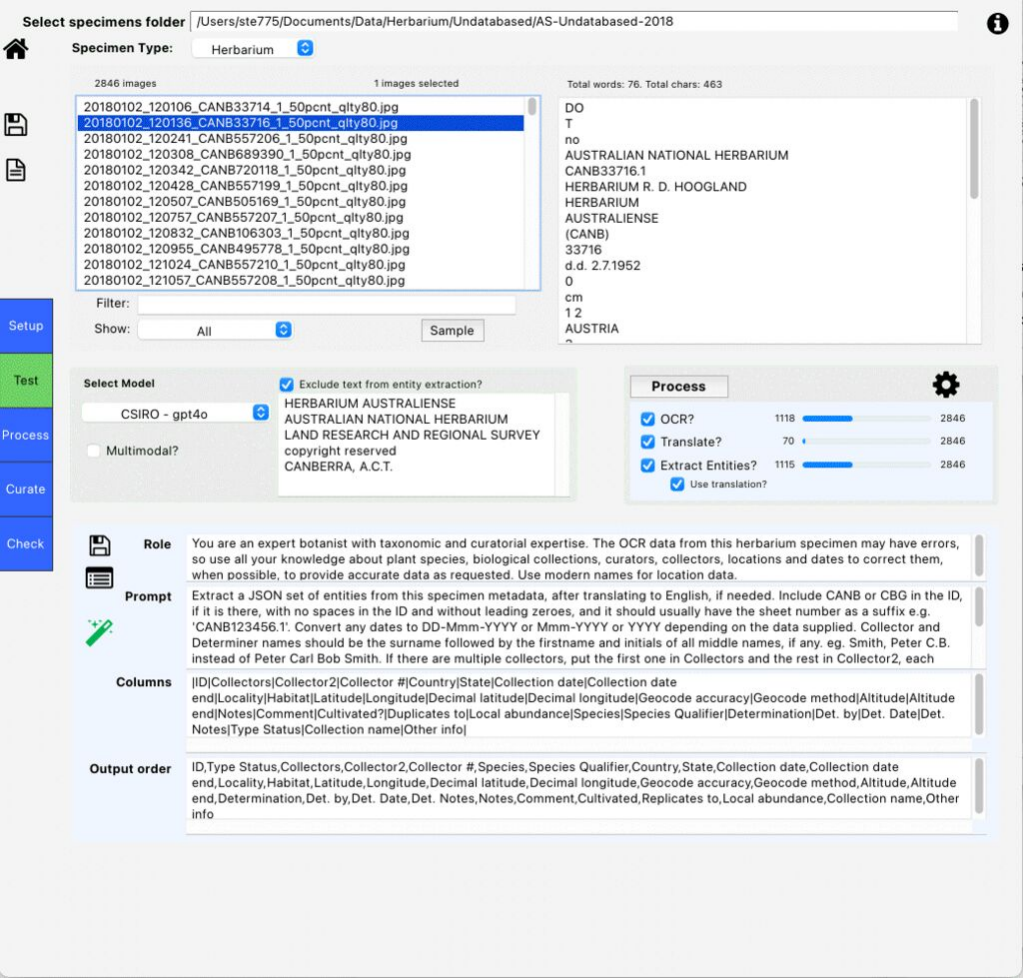
SpeciMate main processing screen.

The main processing screen (see Fig. 1) provides the core functionality for setting up and executing metadata extraction. The main processing workflow follows these steps:

1. Select a folder containing specimen images;
2. Choose the specimen type (herbarium sheet, insect slide, insect type etc.);
3. Select processing options (OCR, Translation, Metadata Entity Extraction);
4. Configure AI models and prompts;
5. Process selected images individually or in batch;
6. Check and correct specimen data using metadata curation screens;
7. Finally, full dataset curation, visualisation and dataset export.

During project setup, steps 4 and 5 will be repeated multiple times to test prompts and processing options on small numbers of specimens to improve results. When these satisfy expectations, then all images in a folder can be rapidly processed, before subsequent manual checking and curation occurs in steps 6 and 7.

The main screen displays processing progress and status, including counts of processed specimens for each operation. The list of images in the folder can be filtered by text string or to show or exclude images that have already been processed by OCR or LLM, to enable selective processing. SpeciMate includes an image viewer that allows users to examine specimens during processing and curation. The viewer supports zooming, panning, rotation and the display of OCR-detected text blocks.

*Metadata Curation*

**Figure 2.**
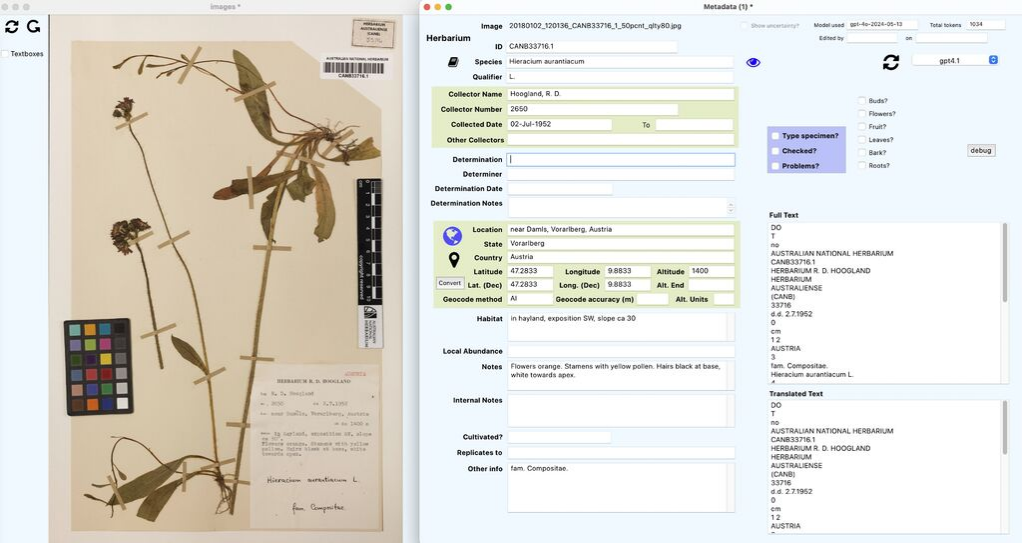
Metadata curation screen and image viewer.

A metadata curation screen, as illustrated in Fig. 2, enables users to edit the extracted metadata fields while viewing the specimen image, providing essential contextual information. The curation interface offers editable fields for all extracted metadata elements and easy navigation between specimens. The user can reprocess a specimen with a different AI model, if desired. In addition, the system integrates with various external services for verification purposes. These integrations include species name validation against the Atlas of Living Australia (ALA - https://www.ala.org.au/) (Belbin et al. 2021), Global Biodiversity Information Facility (GBIF - https://www.gbif.org/) or Global Names Architecture (GNA - https://globalnames.org/) webservices (Pyle 2016); location verification using Google Maps (https://www.google.com/maps); geocoding of locality text, if desired, using the Nominatim web service (https://nominatim.openstreetmap.org/ui/search.html); and conversion of verbatim coordinates in a variety of formats to decimal format for standardisation.

The interface also includes special check-boxes for specimen-specific attributes (e.g. type specimen status, presence of buds/flowers/fruit for herbarium specimens). Another check-box marks that these metadata have been human-curated and, if selected, locks this record so that it cannot be re-processed inadvertently. This provides a means of checking which records have been curated and which data have only been processed by AI. Lastly, there is a check-box for flagging specimens which may contain issues for attention by a human curator, along with a text input area for entering details on these.

*Dataset Curation*

**Figure 3.**
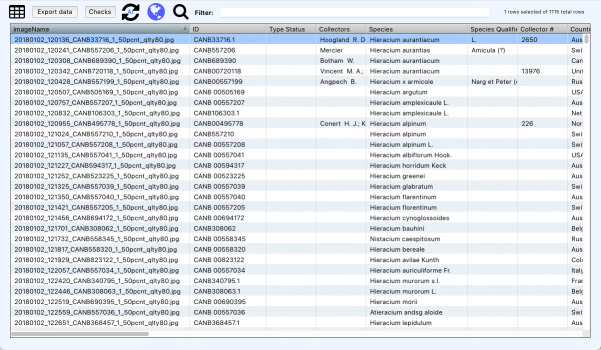
Dataset curation tabular screen, enabling quick consistency and error checking.

**Figure 4.**
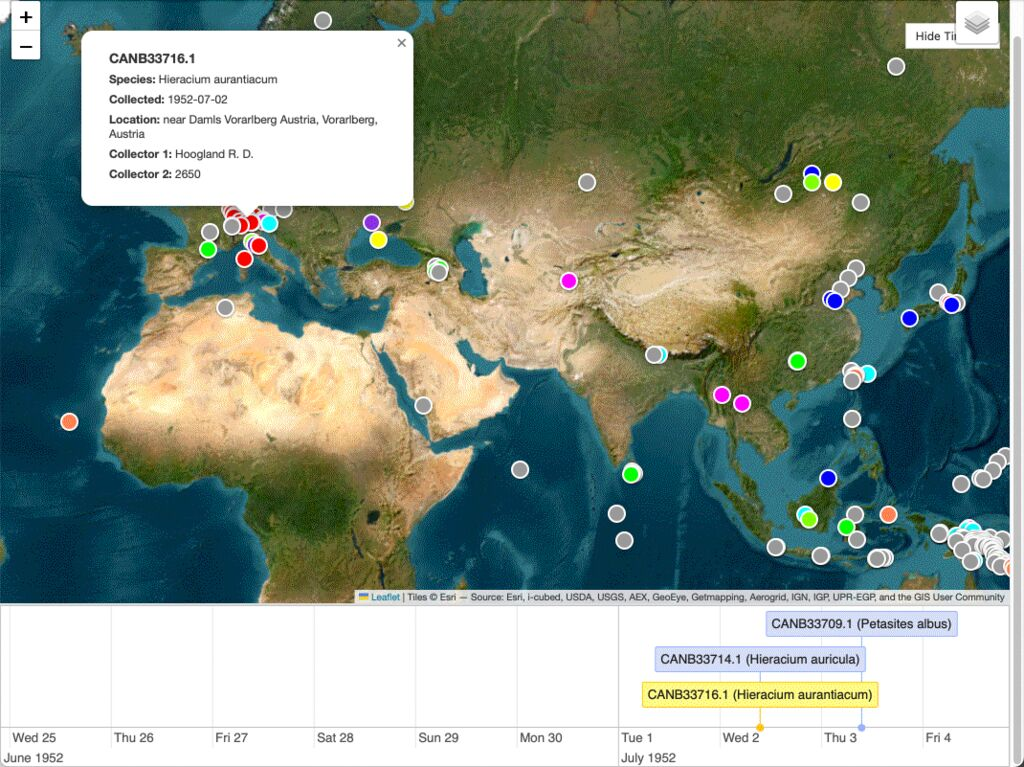
Dataset map and timeline screen for detecting potential spatial and temporal patterns and outliers within a dataset.

SpeciMate provides a screen for reviewing and managing the entire dataset through a tabular interface (Fig. 3). Users can sort, filter or search the dataset to group related specimens or to locate specific ones. This can help to identify and select specimens with potential errors that require attention, such as unexpected blanks, formatting errors or other extraction or OCR transcription errors, such as misspelling or LLM “hallucination”. Users can run automated checks, such as identifying ID/filename mismatches and reprocess selected specimens when needed. The dataset curation screen also includes a visualisation tool that displays specimen collection locations on a map and collection dates on a timeline (Fig. 4), helping to identify spatial and temporal patterns or outliers within the collection data. Map markers can also be moved on the map to interactively modify the underlying location data. Users can finally export the dataset to tab-separated values (TSV) format for import into their collection management or other system.

*Prompt Management*

**Figure 5.**
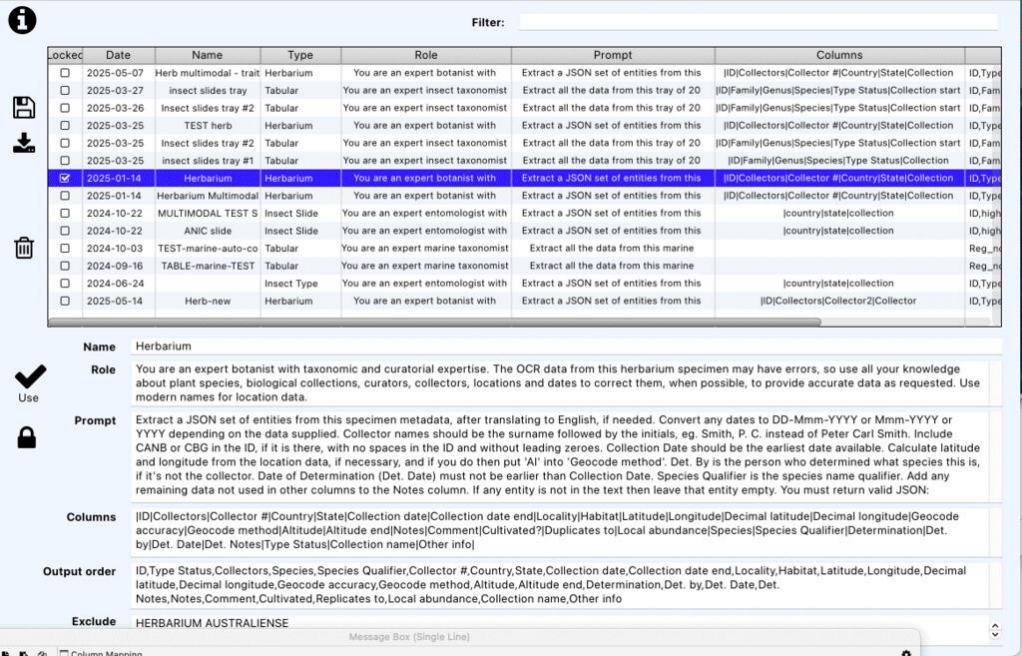
Prompt Management screen showing available prompts with details for the selected prompt.

SpeciMate includes a comprehensive prompt management system, which allows users to create, edit, save and share prompts for different specimen types and extraction tasks (Fig. 5). The prompt management interface enables several key functions. Users can select existing prompts from an established library of templates. They can edit various prompt components, including role definitions, prompt text, column specifications and other parameters. The system allows for locking prompts to prevent unintended modifications, thereby preserving validated configurations. Additionally, users can export and import prompts in JSON format to facilitate sharing across different platforms and projects. This functionality may support the creation of a knowledge base of effective prompts that can be refined over time and shared across different digitisation projects both within and amongst organisations, leading to collaborative improvement of data extraction techniques.

*Service Configuration*

SpeciMate allows users to add and configure the AI services used for OCR, translation and entity extraction. The service configuration interface enables the creation and modification of these services and associated parameters, such as API URL, API key, URL request template and request and token limits. This provides some flexibility to adapt to changes in AI services and integrate new services and models as they become available. Using LLM models running on a local machine is also possible with the only restriction being that the system uses openAI API formats. This was tested successfully using the LM Studio application (https://lmstudio.ai/) to host a local LLM (Phi-3.1-mini-4k-instruct-Q4_K_M.gguf) on a Macbook Pro with M1 Pro chip and 16 GB memory running macOS 13.7.5.

*Logging*

Logging within SpeciMate occurs during specimen processing and curation, with OCR, translation and metadata extraction processes logged with timestamps so performance can be evaluated and any errors noted. Metadata editing processes are logged to a file on a per-field basis along with timestamps which provides some audit trail functionality and a basis for evaluating performance. For example, if desired, the log files could be analysed to see which fields often need editing, how long each specimen takes to curate, how long each processing step takes and so on.

**Sample Outputs**

Here, we present sample outputs from processing herbarium and insect specimens, without any user curation of the data.

*Insect specimens*

Table 2 shows the results from processing the sample insect specimens in Fig. 6 and illustrates some of the power and interesting capabilities of the application. Image "a" demonstrates both the difficulties that OCR processes may have with hard-to-interpret handwriting and also the power of using a LLM to interpret and correct such errors during extraction. For example, the City of *Townsville* in Queensland, Australia is corrected by the LLM from the original OCR text of *Townsvilk*, similarly the collector name. Image "b" demonstrates how well a multimodal LLM can perform on multiple typed text labels. In this case, it correctly extracts the specimen host details from a label, while, in a prior test, the OCR process had recognised the host genus *Thryptomene* as a continuation of the location line, resulting in *Mt. Webb Thryptomene* in one line, resulting in a subsequent error from the LLM.

*Herbarium Specimens*

Table 3 shows results from processing three sample herbarium specimens in Fig. 7a, b, c. Sample "a" demonstrates results from using a multimodal model (openAI GPT-4.1) to extract the data without the OCR process, correctly extracting and formatting almost all requested columns, with notable errors on the hard-to-read handwriting on determiner name and determination date. Samples "b" and "c" demonstrate the accuracy of the OCR process and of the entity extraction process for both typed and handwritten labels. Note that the OCR has picked up the latitude and longitude from faint pencil-writing on the lower-left corner of sample "b", while the LLM has correctly excluded extraneous text resulting from subsection labels that have been crossed out in the original and incorrectly extracted in the OCR process. Sample "c" demonstrates the LLM correcting an OCR error for the determiner name: OCR extracted “A.D. Poulan” which the LLM corrected and formatted to “Poulsen, A.D.”. This sample also shows the LLM inferring approximate latitude and longitude from the location description, which may assist subsequent curation using the mapping features.

*Processing Time and Cost*

The time taken to process specimens can vary greatly and is primarily dependent on the models and infrastructure used. For example, we processed 100 herbarium specimens with OCR and entity extraction using the openAI gpt-4.1 LLM model. This took 83 seconds and cost $0.42 for the LLM and $0.15 for the OCR (note that 1000 images are free per month using Google Vision OCR and then cost $1.50 per 1000 images).

**Discussion**

The development and use of SpeciMate shows how human-AI collaboration can effectively extract metadata from biological specimens through two key phases: prompt engineering, where human domain expertise guides AI models to extract metadata of interest and data curation, where human review ensures accuracy, while AI models provide scalable pattern recognition. This complementary workflow leverages AI's processing capabilities alongside human contextual understanding and quality judgement, resulting in a metadata extraction approach that surpasses both manual transcription efficiency and fully automated accuracy. Our testing and use of SpeciMate shows several interesting capabilities and areas for further development.

*Error Correction and Contextual Understanding*

High-quality Large Language Models for entity extraction demonstrate capabilities to correct errors resulting from OCR process difficulties when transcribing specimen labels. These include spelling corrections, reconstruction of text order when OCR incorrectly detects lines or columns and recognition of entity relationships even when spatially separated on labels. This probably derives from the models' broad understanding of language and domain knowledge about biological specimens, enabling inferences beyond simple text extraction when provided with prompts that supply contextual information and guidance.

*Contextual Inference*

Large language models are often able to successfully infer missing metadata from the available context. For example, they can derive country or state names from locality descriptions provided on specimen labels. These models can also determine taxonomic hierarchy information, such as family and genus classifications, from species names alone. Additionally, LLMs can generate approximate geographic coordinates from textual locality descriptions, converting verbal geographic references into numerical data. This inferential capability adds significant value by enriching the extracted metadata beyond what is explicitly stated on specimen labels, if desired, potentially reducing other curation tasks downstream. Even approximate location coordinates may assist human curators by providing a starting location which can be further refined using built-in mapping functionality, thereby streamlining the geocoding process.

*Translation Capabilities*

While LLMs can also translate between languages, we find that a specialised translation service like Google Translate currently provides superior results for label text. However, LLM translation may be sufficient for some use cases, potentially simplifying the processing pipeline and reducing costs.

*Hallucinations and Other Errors*

Errors do occur, especially when combining inaccuracies in the OCR process with LLM-based extraction. LLMs occasionally produce hallucinations or systematic errors, such as inserting generic placeholders as a “best guess” (e.g. "John Doe" for collector names) or returning identical incorrect values across multiple specimens. These errors are now very infrequent when using high-quality LLMs and are usually easily identifiable and corrected during the curation phase. A temperature setting of 0 is used with openAI LLMs to reduce the likelihood of random results (or “hallucinations”) during the metadata extraction process. Full dataset curation using the tabular dataset view can be very helpful to identify repeating errors, which can usually be resolved by reprocessing with adjusted prompts or different models. Other errors can sometimes be harder to identify, especially those resulting from OCR of problematic handwriting; thus, the importance of human curation and checking remains, both at the individual specimen level and at the dataset level.

*Multimodal Capabilities*

Multimodal models that can process images directly show promise for simplifying the workflow by combining OCR, translation and entity extraction in a single pass. These models may better leverage visual context, such as label layouts, which can be useful in cases where the OCR has not produced well-grouped results. If specimens contain symbols that are not handled by OCR, then the multimodal model may also be useful in extracting these. Note that the prompts and templates used for multimodal LLMs will differ since image data are sent to the model rather than just text.

*Cost/Performance trade-offs*

Using powerful, commercial AI services comes with costs which may be a barrier for some organisations. While these may be significant for large numbers of specimens, we expect that the overall process speed-up and effort saved should offset this. For specimens with multiple, complex labels, using a better, more expensive LLM is usually preferred to a cheaper model, as the costs involved per specimen are so low that the time saved through reduced transcription and error correction seems worth it. For example, using openAI’s high-quality GPT-4.1 model is usually preferred to the GPT-4.1-mini model, although the faster and cheaper “mini” model may be quite usable in some cases. To reduce costs further, there are an increasing number of freely available models which may be accessed through sites such as HuggingFace (www.huggingface.com). If there are locally available machines capable of running some open-access LLMs, then these can be downloaded and set up to be freely accessed. Note that quality may be affected and processing time may increase as multiple concurrent requests sent to a local machine may not be possible. The author (AS) has successfully used a locally hosted LLM on an Apple Macbook Pro as described earlier, though quality and speed were both lower in comparison to the cloud-based services. Note, however, that LLM model development is a rapidly changing space, so high-quality models running locally which satisfy some purposes will soon be available or already are.

**Future Work**

Based on our experience with SpeciMate and feedback from users, we have identified several directions for future development.

*Flexibility of Specimen Definition*

Work to enable dynamic specimen definition is needed to provide more flexibility. This will enable any columns to be extracted and curated, with the data curation screens being dynamically created according to the column specifications. Each specimen type may present unique metadata requirements that could require prompt engineering and potentially new interface components. We have also started working on integrating the dynamic definition of pick-lists to constrain possible values for selected columns. These could be defined in a text file or spreadsheet, which might be extracted from a collections database system and imported into the future metadata definition interface. The ability to link to and use existing ontologies to guide and define metadata specifications may also be useful.

*Label Detection and Processing*

Future versions of SpeciMate could incorporate label detection models to automatically identify and prioritise relevant labels on specimen images (Thompson et al. 2023). This may help address challenges with complex specimens that contain multiple labels of different ages, purposes and relevance, such as specimens that have been re-identified multiple times. Prior work indicates this can provide useful performance improvements (Turnbull et al. 2024), although it may also be unnecessary as multimodal models improve and label characteristics can be detected through natural language prompts.

*Integration of Species Identification and Trait Extraction Models*

The ability to add specialised computer vision models to the system would provide potentially huge gains to the future capabilities of the application. If we could provide support for including this as an option in the workflow, this may enable automated species recognition or trait extraction. Species recognition would assist with identifying not-yet identified species within a collection or where species have been mis-identified or where species need updating. Trait extraction capabilities would provide extra information from a specimen, such as leaf or wing dimensions or the presence or absence of features such as flowers or seeds and other traits. Initial tests have indicated that using multimodal LLMs may provide some additional trait data, albeit with limitations around accuracy. SpeciMate can support the initial testing of these ideas through being able to define new services combined with suitable prompt definitions.

*Extended Integration with External Services*

Future development could also expand integration with biodiversity databases and services, including direct validation against taxonomic authorities beyond ALA and GBIF, enhanced geocoding and geographic validation services and integration with collector databases for name validation. This would further enhance data validation capabilities and streamline the workflow from extraction to database integration.

**Conclusions**

SpeciMate has the potential to improve the digitisation of natural history collections through its innovative human-AI collaborative approach to metadata extraction. By integrating state-of-the-art AI services with interactive human curation tools, SpeciMate addresses the bottleneck of metadata extraction in the digitisation workflow. The application's design emphasises accessibility, flexibility and quality control, making it suitable for a wide range of collection types and institutional contexts. The two-phase collaboration model — prompt engineering during setup and interactive curation after processing — leverages the complementary strengths of human expertise and AI capabilities to achieve results that neither could accomplish alone efficiently.

Our observations of SpeciMate in operation highlight both the impressive capabilities of current AI models in understanding and extracting information from specimen labels and the continuing importance of human oversight and domain knowledge in ensuring accurate results. As AI technologies continue to evolve, applications like SpeciMate will play an increasingly important role in unlocking the vast scientific potential of natural history collections, making these invaluable resources more accessible for research, education and conservation efforts worldwide.

## Web location (URIs)

Homepage: https://github.com/alanstenhouse/SpeciMate

## Technical specification

Programming language: LiveCode, Javascript

Operational system: MacOS, Windows

## Repository

Type: Git

## Usage licence

### Usage licence

Other

### IP rights notes

CSIRO Non-Commercial License (based on BSD-3-Clear)

## Figures and Tables

**Figure 6a.**
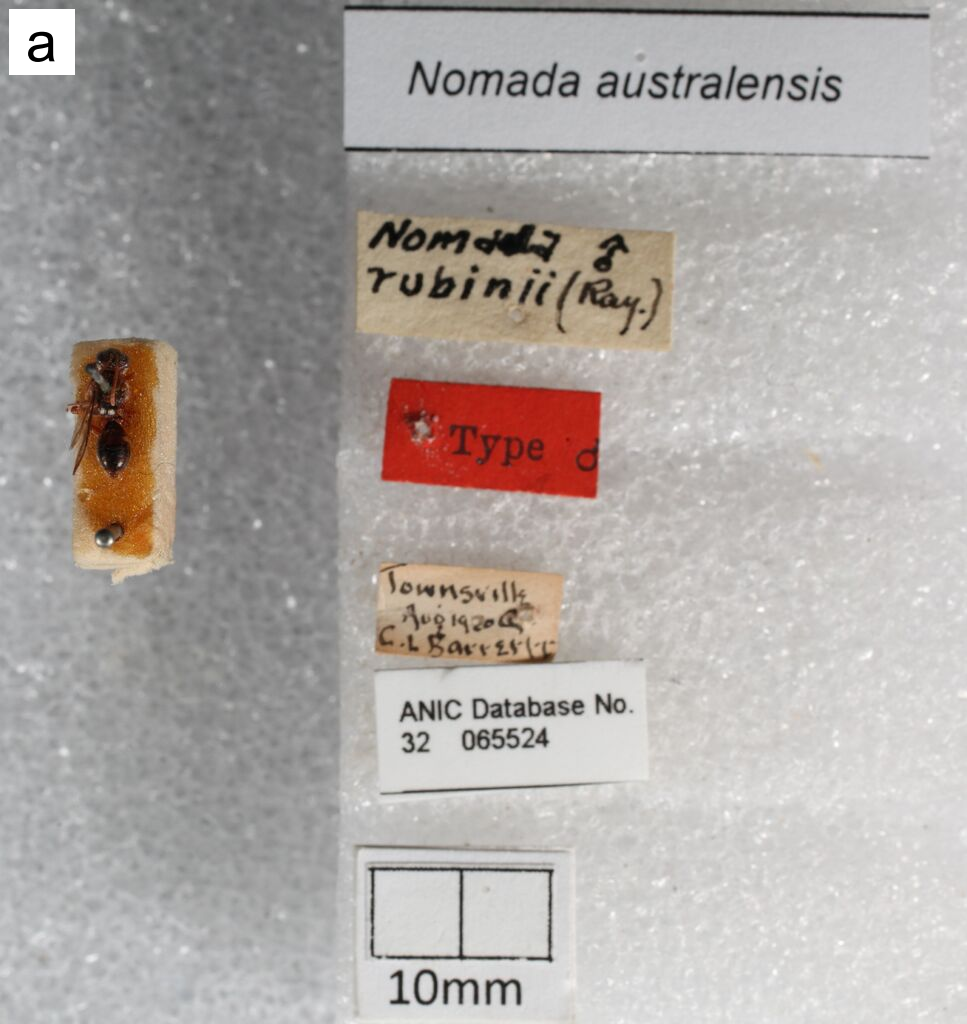


**Figure 6b.**
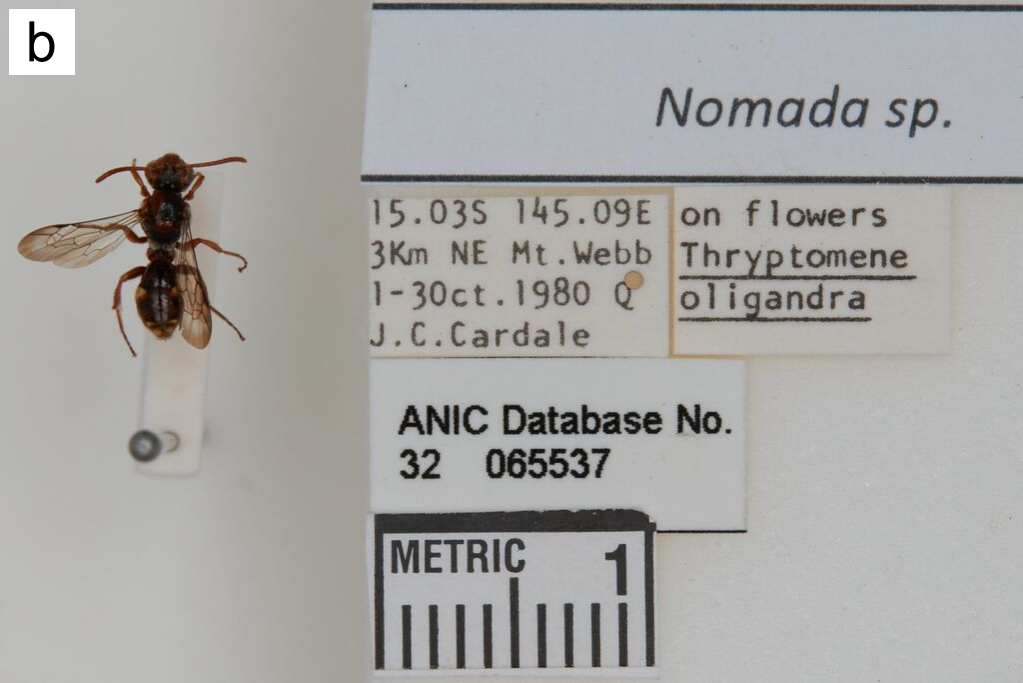


**Figure 7a.**
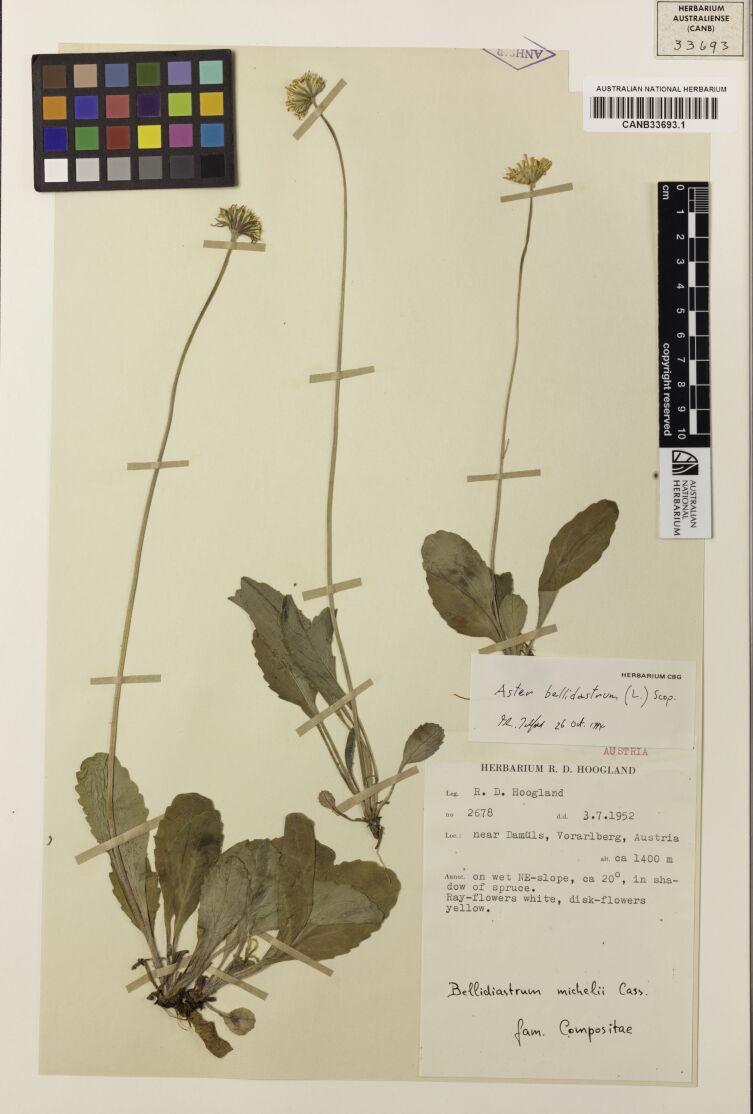


**Figure 7b.**
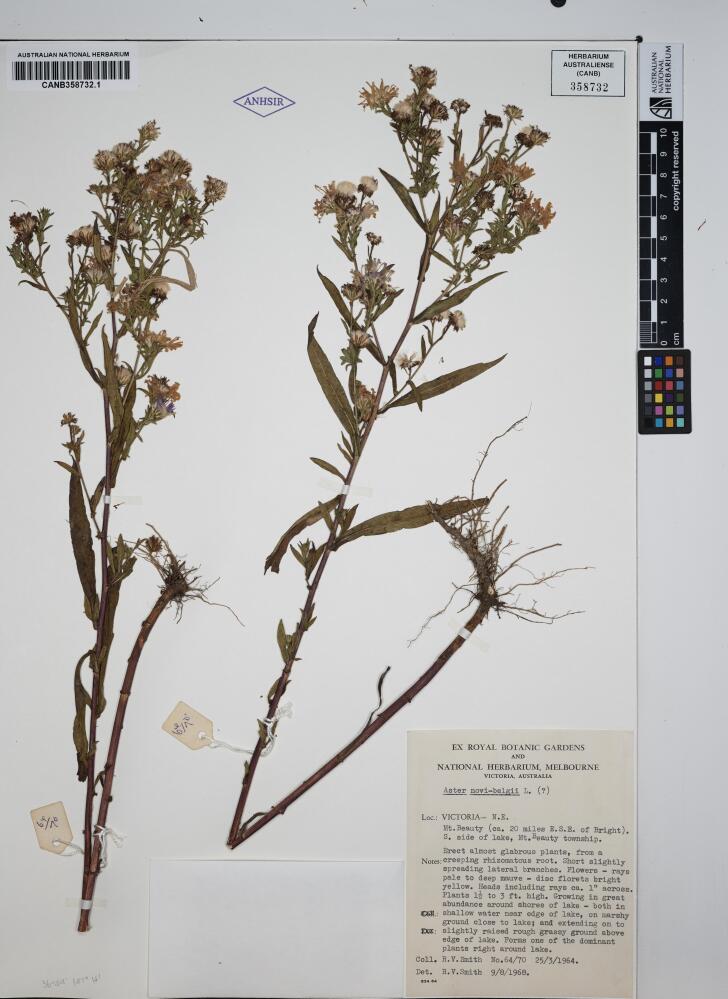


**Figure 7c.**
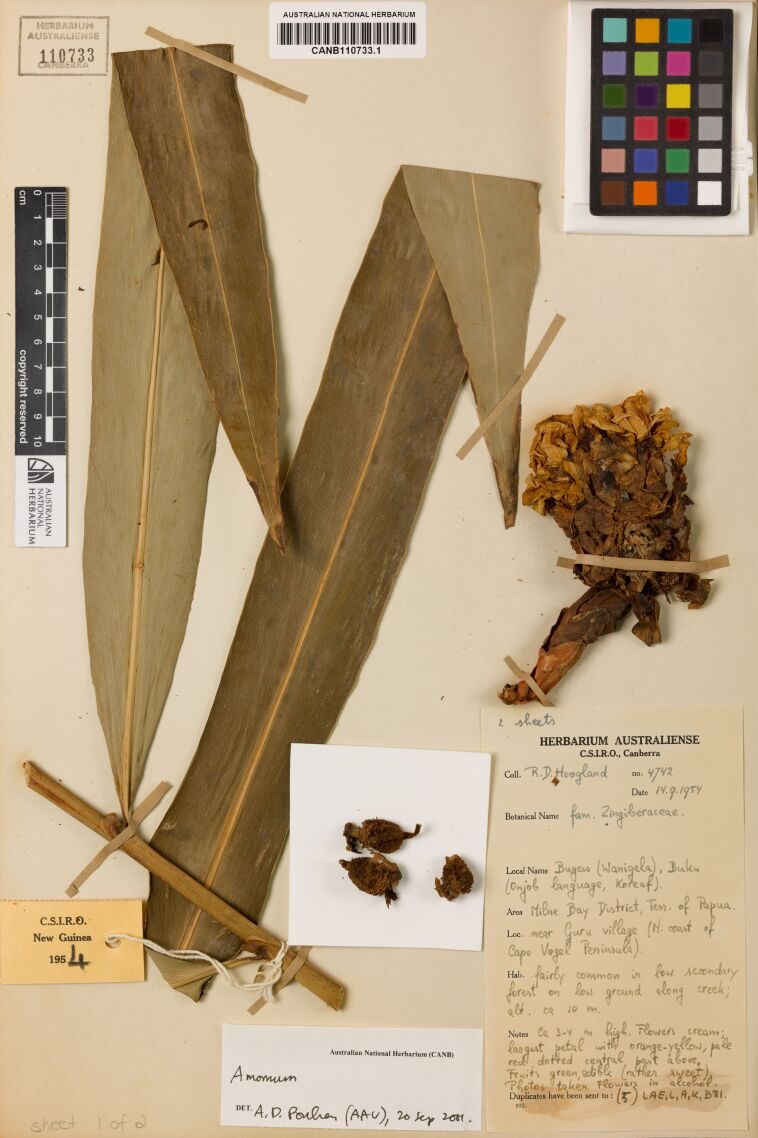


**Table 1.** Example LLM prompt details to extract metadata from herbarium specimens, including column specifications and formatting instructions, for use in the SpeciMate application.

Role	You are an expert botanist with taxonomic and curatorial expertise. The OCR data from this herbarium specimen may have errors, so use all your knowledge about plant species, biological collections, curators, collectors, locations and dates to correct them, when possible, to provide accurate data as requested. Use modern names for location data.
Prompt	Extract a JSON set of entities from this specimen metadata, after translating to English, if needed. Convert any dates to DD-Mmm-YYYY or Mmm-YYYY or YYYY depending on the data supplied. Collector names should be the surname followed by the initials, eg. Smith, P. C. instead of Peter Carl Smith. Include CANB or CBG in the ID, if it is there, with no spaces in the ID and without leading zeroes. Collection Date should be the earliest date available. Calculate latitude and longitude from the location data, if necessary, and if you do then put 'AI' into 'Geocode method'. Det. By is the person who determined what species this is, if it's not the collector. Date of Determination (Det. Date) must not be earlier than Collection Date. Species Qualifier is the species name qualifier. Add any remaining data not used in other columns to the Notes column. If any entity is not in the text then leave that entity empty. You must return valid JSON:
Metadata Columns	|ID|Collectors|Collector #|Country|State|Collection date|Collection date end|Locality|Habitat|Latitude|Longitude|Decimal latitude|Decimal longitude|Geocode accuracy|Geocode method|Altitude|Altitude end|Notes|Comment|Cultivated?|Duplicates to|Local abundance|Species|Species Qualifier|Determination|Det. by|Det. Date|Det. Notes|Type Status|Collection name|Other info|

**Table 2.** Sample insect specimen results.

**Sample**	**OCR output**	**Extracted columns**
a	Nomada australensis Nom drubinii (Ray.) Type o Townsvilk 1920 C.L Barrest ANIC Database No. 32 065524 10mm	ID: 32-065524 Genus: Nomada Species: australensis Type Status: Type Collector: Barrett, C.L. Collected Start: 1920 Location: Townsville State: Queensland Country: Australia Other info: Nomada rubinii (Ray.)
b	*<no OCR text, processed using a multimodal LLM>*	ID: 32-065537 Genus: Nomada Collector: Cardale, Jean C. Collected Start: 01/10/1980 Collected Start: 30/10/1980 Location: 3 km NE Mount Webb State: Queensland Country: Australia Remarks: on flowers Associate type: plant Specimen Association: host Associated Taxon: Thryptomene oligandra

**Table 3.** Sample Herbarium specimen results.

**Sample**	**OCR output**	**Extracted columns**
a	*<no OCR text, processed using a multimodal LLM>*	ID: CANB33693.1 Species: Aster bellidiastrum Collector: Hoogland, R.D. Collector #: 2678 Collected Date: 03-Jul-1952 Determination: Aster bellidiastrum (L.) Scop. Determiner: Zaglmayr, H. Determination Date: 26-Oct-1974 Location: near Damüls, Vorarlberg, Austria State: Vorarlberg Country: Austria Altitude: 1400 Latitude (Dec): 47.2797 Longitude (Dec): 9.8881 Geocode method: AI Habitat: on wet NE-slope, ca 20°, in shadow of spruce Notes: Ray-flowers white, disk-flowers yellow. Other info: Bellidiastrum michelii Cass.; fam. Compositae
b	AUSTRALIAN NATIONAL HERBARIUM CANB358732.1 358732 ANHSIR EX ROYAL BOTANIC GARDENS AND NATIONAL HERBARIUM, MELBOURNE VICTORIA, AUSTRALIA Aster novi-belgii L. (?) 6/10 36°49' 147° 14' Loc.: VICTORIA- N.E. Mt. Beauty (ca. 20 miles E.S.E. of Bright). S. side of lake, Mt. Beauty township. Erect almost glabrous plants, from a Notes: creeping rhizomatous root. Short slightly spreading lateral branches. Flowers rays pale to deep mauve disc florets bright yellow. Heads including rays ca. 1" across. Plants 1 to 3 ft. high. Growing in great abundance around shores of lake both in Co: shallow water near edge of lake, on marshy ground close to lake; and extending on to Da: slightly raised rough grassy ground above edge of lake. Forms one of the dominant plants right around lake. Coll. R. V. Smith No.64/70 25/3/1964. Det. R.V. Smith 9/8/1968. 834.64	ID: CANB358732.1 Species: Aster novi-belgii Qualifier: (?) Collector Name: Smith, R.V. Collector Number: 64/70 Collected Date: 25-Mar-1964 Determination: Aster novi-belgii L. (?) Determiner: Smith, R.V. Determination Date: 09-Aug-1968 Location: Mt. Beauty (ca. 20 miles E.S.E. of Bright), S. side of lake, Mt. Beauty township State: Victoria Country: Australia Latitude: 36°49′ Longitude: 147°14′ Lat. (Dec): –36.8167 Long. (Dec): 147.2333 Geocode method: AI Habitat: Shallow water near edge of lake, on marshy ground close to lake; extending onto slightly raised rough grassy ground above edge of lake. Forms one of the dominant plants right around lake. Local Abundance: Great abundance around shores of lake Notes: Erect almost glabrous plants, from a creeping rhizomatous root. Short slightly spreading lateral branches. Flowers rays pale to deep mauve, disc florets bright yellow. Heads including rays ca. 1'' across. Plants 1 to 3 ft. high. Growing in great abundance around shores of lake both in shallow water near edge of lake, on marshy ground close to lake; and extending onto slightly raised rough grassy ground above edge of lake. Other info: Ex Royal Botanic Gardens and National Herbarium, Melbourne
c	HERBARIUM AUSTRALIENSE 110733 CANBERRA AUSTRALIAN NATIONAL HERBARIUM CANB110733.1 New Guinea 195. 4 2 sheets HERBARIUM AUSTRALIENSE C.S.I.R.O., Canberra Amomum Australian National Herbarium (CANB) DET. A.D. Poulan (AAU), 20 Sep 2001. Coll. R.D. Hoogland Botanical Name no. 4742 Date 14.9.1954 fam. Zingiberaceae. Local Name Bugew (Wanigela), Buku (Onjob language, Koreaf). Area Milne Bay District, Terr. of Papua. Loc. near Guru village (N. count of Cape Vogel Peninsula). Hab. fairly forest common in low secondary on low ground along creek; alt. ca 10 m. Notes Ca 3-4 m high. Flowers cream; largest petal with orange-yellow, pale red dotted central part above, Fruits Photos taken. Flowers in alcohol. Duplicates have been sent to: (5) LAE, L, A, K, BRI. green, edible (rather sweet, 932. sheet 1 of 2	ID: CANB110733.1 Species: Amomum Collector Name: Hoogland, R.D. Collector Number: 4742 Collected Date: 14-Sep-1954 Determiner: Poulsen, A.D. Determination Date: 20-Sep-2001 Determination Notes: (AAU) Location: near Guru village (N. count of Cape Vogel Peninsula), Milne Bay District, Terr. of Papua State: Milne Bay Country: Papua New Guinea Latitude: –10.3167 Longitude: 150.3333 Altitude: 10 Lat. (Dec): –10.3167 Long. (Dec): 150.3333 Geocode method: AI Habitat: fairly common in low secondary forest on low ground along creek Notes: pale red dotted central part above. Fruits green, edible (rather sweet). Photos taken. Flowers in alcohol. Duplicates have been sent to: (5) LAE, L, A, K, BRI. Replicates to: LAE, L, A, K, BRI Other info: 2 sheets
